# Supplementary material for: Intraoperative Assessment of Tumor Margins in Tissue Sections with Hyperspectral Imaging and Machine Learning
Source: Cancers (Basel). 2022 Dec 29;15(1):213. doi: 10.3390/cancers15010213 (PMC9818424; doi:10.3390/cancers15010213)
Supplement: Supplementary file 1 [file cancers-15-00213-s001.zip › cancers-2074685-supplementary.pdf]

Supplementary

Table S1. No. of slides and tiles per patient.

Patient	No. of Slides	Used tiles per slide	Total no. of tiles
1	3	136, 111, 55	301
2	2	80, 117	197
3	2	64, 36	100
4	6	110, 164, 164, 191, 180, 118	927
5	4	203, 234, 265, 265	967
6	3	125, 240, 243	608
7	3	110, 132, 139	381

Table S2. No. of ‘tumor’ and ’healthy’ tiles in each fold.

Fold	Validation set	No. of healthy tiles in validation set	No. of tumor tiles in validation set	No. of healthy tiles in training set	No. of tumor tiles in training set
1	Patient 5 Slide 3, Patient 4 Slide 6, Patient 7 Slide 3, Patient 6 Slide 3, Patient 1 Slide 2	143	427	419	1205
2	Patient 6 Slide 2, Patient 5 Slide4, Patient 2 Slide 2, Patient 4 Slide 2, Patient 1 Slide 3	65	427	497	1205
3	Patient 7 Slide 3, Patient 2 Slide 2, Patient 4 Slide 3, Patient 5 Slide 3, Patient 3 Slide 2	175	302	387	1330
4	Patient 3 Slide 1, Patient 2 Slide 1, Patient 5 Slide 3, Patient 6 Slide 1, Patient 4 Slide 1	157	245	405	1387
5	Patient 6 Slide 2, Patient 7 Slide 2, Patient 4 Slide 4, Patient 1 Slide 1, Patient 2 Slide 2	82	444	480	1188
6	Patient 6 Slide 3, Patient 7 Slide 1, Patient 4 Slide 5, Patient 5 Slide 1, Patient 1 Slide 1	110	377	452	1255

Table S3. Tumor classification sensitivity, specificity, accuracy and F1-score.

	Sensitivity	Specificity	Accuracy	F1-Score
ResNet Finetuning on false color images	0.40 ± 0.28	0.92 ± 0.07	0.76 ± 0.11	0.73 ± 0.15
ResNet Finetuning on RGB images	0.21 ± 0.24	0.94 ± 0.07	0.73 ± 0.09	0.66 ± 0.14
3D CNN	0.48 ± 0.24	0.89 ± 0.05	0.76 ± 0.10	0.74 ± 0.14

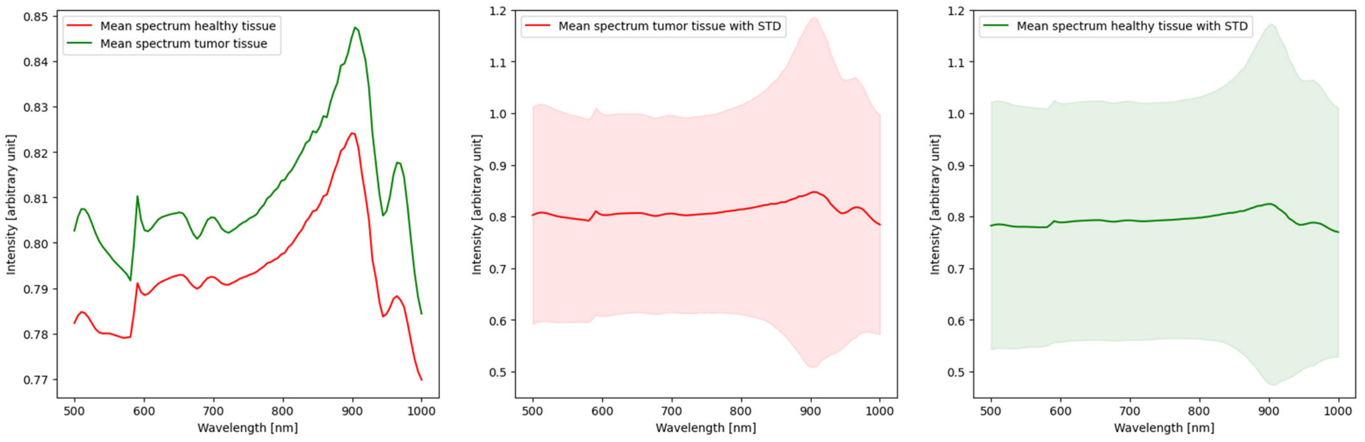

Figure S1. Mean spectra of healthy and tumor tissue. Detailed view with standard deviation of mean spectra.
